# Adequacy of Some Locally Produced Complementary Foods Marketed in Benin, Burkina Faso, Ghana, and Senegal

**DOI:** 10.3390/nu10060785

**Published:** 2018-06-18

**Authors:** Sara Antonio Dimaria, Hélène Schwartz, Christèle Icard-Vernière, Christian Picq, Noël Marie Zagre, Claire Mouquet-Rivier

**Affiliations:** 1 UMR Nutripass, French National Research Institute for Sustainable Development (IRD), Université de Montpellier, SupAgro, 34000 Montpellier, France; sara.antoniodimaria@gmail.com (S.A.D.); christele.verniere@ird.fr (C.I.-V.); christian.picq@ird.fr (C.P.); 2 United Nations Children's Fund (UNICEF), West and Central Africa Regional Office, P.O. Box 29720 Dakar, Sénégal; hschwartz@unicef.org (H.S.); nzagre@unicef.org (N.M.Z.)

**Keywords:** processed cereal-based blends, West-African countries, mineral content, labelling information

## Abstract

Adequate complementary foods are needed to help reduce the high prevalence of stunting in children in many Low and Middle Income Countries (LMICs). We assessed the availability, affordability, and nutrient adequacy of imported and locally produced processed cereal-based blends (PCBBs), marketed as complementary food for young children in Benin, Burkina Faso, Ghana, and Senegal. In total, 19 local producers and 275 points of sale in the four countries were surveyed to evaluate the quantities and accessibility of PCBBs. In addition, 32 PCBBs were analysed for their nutritional composition and packaging information. The results showed that only 7 out of 32 PCBBs could be classified as nutritionally satisfactory. Access to the products was insufficient in all surveyed settings. At the points of sale, the PCBB market was dominated by imported products, even though two out of four imported PCBBs were not nutritionally satisfactory. Imported PCBBs were two to three times more expensive than locally produced PCBBs. Labelling of the PCBBs was inadequate in many aspects. Technical support should be offered to local PCBB producers to ensure the adequate formulation and supply of an appropriate vitamin and mineral premix. The development of national specific regulations on PCBB composition and labelling is strongly recommended in these countries.

## 1. Introduction

Recent anthropometric data on young children in Low and Middle Income Countries (LMICs) show that the prevalence of chronic malnutrition or stunting increases drastically from the age of three months up to 24 months [1]. Despite a decrease in prevalence from 42% in 1990 to 34% in 2013 in sub-Saharan Africa, the actual number of children under five with stunting is still increasing because of population growth [2]. Also, only 10% of children aged 6–23 months in this region have access to a minimum acceptable diet [3]. Yet, one of the conditions required to prevent stunting and its critical consequences is to meet the daily nutritional requirements of young children by introducing adequate complementary foods, starting at the age of six months, and using appropriate complementary feeding practices, while pursuing breastfeeding up to the age of 24 months [4,5].

In addition to homemade meals cooked specifically for young children or for all family members, commercially available processed cereal-based blends (PCBBs), fortified or not, are the main type of complementary foods consumed by young children in developing countries [6]. More specific products fortified with vitamins and minerals, such as lipid-based pastes (ready-to-use foods or RUFs), high-energy biscuits (HEB), or “Compressed Food” Bars, have also been designed to treat or prevent acute malnutrition or stunting. These specific products are, however, rarely available through conventional marketing channels.

It is well recognised that regular consumption of well-formulated fortified PCBBs has a positive impact on the nutritional status of young children in LMICs [7]. PCBBs are usually made from cereals or starchy raw materials to which different sources of proteins and lipids, mainly legumes, but also dried fish or milk powder, micronutrient premix, and other ingredients are added to improve the nutritional value, as well as the taste. When manufactured with locally grown raw materials, locally-produced PCBBs would better meet organoleptic and cultural expectations. These would also benefit the national economy, particularly through the creation of jobs and by providing added value to local raw materials [8]. Almost thirty years ago, Treche and Mbomé [9] investigated the nutritional adequacy of forty cereal-based blends commercialised as complementary foods in African countries and Vietnam, and pointed out their poor nutritional quality. Since, several studies have also investigated some aspects of the nutritional composition of complementary foods marketed in sub-Saharan African and Asian countries [10,11,12], and concluded that most of them were inadequate. 

A preliminary informal study carried out in the region of West Africa allowed us to identify more than 100 different locally produced PCBBs. Based on this first appraisal, the supply in locally produced PCBBs was further investigated and characterized in four West African countries, i.e., Benin, Burkina Faso, Ghana, and Senegal, where stunting rates are still considerable (34%, 19%, 27%, and 17%, respectively) [13]. The aim of the present study was to provide new data on the overall nutritional quality and adequacy, in comparison to international recommendations, of PCBBs commercialised in these four West African countries, in addition to the recent work of Masters et al. [12] and to characterise the availability and accessibility of the products. To this purpose, surveys were conducted with the two main actors of this market: Producers and points of sale (POS), and samples of PCBBs were collected for evaluating their proximate and mineral composition and labelling. 

## 2. Materials and Methods 

### 2.1. Survey of Producers and Points of Sale 

Surveys were carried out in selected areas of Benin, Burkina Faso, Ghana, and Senegal among producers and POS (two key actors in the locally produced PCBBs’ market). These areas were conveniently selected: Cotonou and Bohicon in Benin, Ouagadougou in Burkina Faso, Lekma, La Dade Kotopon, Ada East, and Ada West in Ghana, and Mbour and Tivaouane in Senegal. The surveyed areas represented important portions of the populations, even though the information gathered was not representative of the whole countries. Data were collected using two questionnaires, one administered to local producers and the other one to sellers at the POS. The questionnaire administered to producers aimed at collecting information on the raw materials or ingredients used, the manufacturing processes and the standards used, the average quantities of PCBB produced, and any difficulties encountered in production. Questions asked of the POS focused on the brand of PCBB sold and the prices. The questionnaires were administered to 20 producers (6 in Benin, 6 in Burkina Faso, 6 in Ghana, and 2 in Senegal). The information collected on the six Burkinabe producers was provided by collaborators from the Nutrifaso Programme of the non-governmental organisation, GRET (Professionnels du développement solidaire), a programme which provides support to those six production units. The producers of PCBBs distribute their products in six main types of POS: Pharmacies, supermarkets, grocery shops, petrol stations, neighbourhood shops, markets, and, sometimes, health centres [10]. In total, 323 POS were surveyed in the areas cited above. The data from the POS in Burkina Faso were provided by collaborators from GRET. The study sample was then classified considering the following four types of POS: 66 pharmacies, 108 food supply stores (grouping supermarkets, grocery shops, and markets), 121 neighbourhood shops, and 28 petrol stations. 

### 2.2. Sampling for Nutritional Composition Analyses and Assessment of Adequacy

Thirty-two samples of PCBBs—8 from Benin, 10 from Ghana, 3 from Senegal, 7 from Burkina Faso, and 4 industrial products imported or produced in West Africa under the license from a multinational enterprise and collected in Ghana and in Senegal—were taken from the surveyed POS to determine their proximate and mineral composition and collect information provided on the packaging. A sample of local products were collected when it was sold in at least three different POS in a given area. A second criterion of selection was that the packaging should exhibit enough information i.e., a label with, at least, the name and contact information of the producer and the composition of ingredients. Among products imported or produced under license of a multinational enterprise, only the most frequent ones were sampled for comparison purposes. As described in the Codex Alimentarius (Codex CAC/GL8 1991 rev. 2013 [14] and Codex Stan 074, 1981, rev. 2006 [15]—a more recent revision of the latter, with specific recommendations for products targeting underweight children was drafted in 2013, but, at the time of writing, was not yet fully approved), PCBB destined for 6–23-month-old children should be basically composed of cereal products, such as wheat, rice, barley, oats, maize, millet, and sorghum. Blends may also contain legumes or oil seeds in smaller proportions as they provide proteins and lipids in substantial quantities. A vitamin and mineral premix can also be added to better meet World Health Organization (WHO) recommendations and, hence, infants’ and young children’s nutritional needs. Information on the ingredients used in the composition of the PCBBs was gathered from the producers and from the packaging of the 32 products collected at the points of sale surveyed.

### 2.3. Analytical Methods 

Biochemical analysis was carried out on the 32 samples. The dry matter (DM) content of collected samples was determined by oven-drying at 105 °C to a constant weight. 

After extraction in petroleum ether, the lipid content was determined after acid hydrolysis using the semi-automatic 2055 Soxtec system (Foss, Nanterre, France) according to the 2003.05 and 2003.06 AOAC procedures [16]. 

Protein content (N × 6.25) was determined by the method of Kjeldahl (ISO 1871:2009).

Mineral contents were analyzed by inductively-coupled plasma optical emission spectroscopy using an ICP-OES 5100 (Agilent Technologies, Les Ulis, France) after wet mineralization of 0.4 g sample in H_2_O_2_ and concentrated HNO_3_ (1/7; *v*/*v*) in an Ethos 1 microwave digester (Milestone, Sorisole, Italy). 

All analyses were done in duplicate and values were averaged.

Data management and statistical analysis: Survey data were captured in an Excel spreadsheet and then checked carefully. Responses to open-ended questions were grouped and coded according to the main trends and ideas. SPSS Software (IBM corporation, Chicago, United States of America) was then used for descriptive analysis.

## 3. Results

### 3.1. Quantities of Locally Produced PCBBs: Availability

Using 2016’s age pyramids, the total number of 6–23 month-old children requiring adequate complementary foods in the four countries was estimated (Table 1). 

Then, the quantities of complementary foods required to feed these populations based on the consumption of 50 g of PCBB/child/day were estimated at, roughly, 800 to 2000 tons/month/country, according to the country. This daily ration size of 50 g of PCBB was suggested as being reasonable by Lutter & Dewey [18]. Local producers of PCBBs were, generally, small and medium-sized enterprises, community or family enterprises, women’s groups or associations, and, more rarely, industrial-scale enterprises. As mentioned above, questionnaires were administered to 20 producers who were classified in three categories (Table 2). These categories were based on the quantities of PCBB they produced and the corresponding number of infants and young children (IYC) that could be nourished, assuming a daily consumption of 50 g. Thus, 1.5 ton of PCBB could feed up to 1000 breastfed infants and young children for one month. Producers in Class I produced more than 3 tons, a quantity that could nourish more than 2000 IYC. Producers in Class II and III produced, respectively, 0.6 to 3 tons/month (corresponding to 400–2000 IYC) and less than 0.6 ton (less than 400 IYC). Among the producers having large production units (Class I), three were located in Benin, one in Ghana, two in Burkina Faso, and one in Ivory Coast. Misola burkinabè association in Class I was based on a different model, consisting of the multiplication of small production units in different settings. Since the 1980s, when this concept was created, production units have boomed in several African countries and total production is currently significant. This method of production has the advantage of keeping production means simple, with little maintenance required, meaning small production units could function successfully in remote rural areas. Class II and Class III grouped, respectively, 7 and 6 producers. Producers in Class I were industrial or semi-industrial enterprises who had the resources to apply more sophisticated processing, such as extrusion-cooking, which generally requires expensive equipment that small enterprises could not afford. The only four producers who exported their products were also in Class I. In Class III, production means were very simple and community facilities were often used to perform processing steps, such as dehulling or milling. Producers in Class III did not add any mineral and vitamin premix (Table S1).

### 3.2. Prices and Budget Required to Purchase PCBBs: Accessibility and Affordability

The mean price of the products corresponding to each sample collected was estimated based on the data collected during the surveys at the POS (Figure S1). Imported PCBBs were much more expensive, with prices ranging from 6.5 to 10.9 euros/kg, than locally processed products whose prices ranged from 2.0 to 6.8 euros/kg. Yet, imported PCBBs had a leading position on the market, notably, in Burkina Faso, Ghana, and Senegal, where nearly all the surveyed distributors sold imported products and the number of imported products on sale at a given POS was higher than the number of locally produced PCBBs. Among the 963 product items identified in the 275 surveyed POS, 646 (67.1%) were imported PCBBs against 317 (32.9%) PCBBs locally produced.

**Figure 1:**
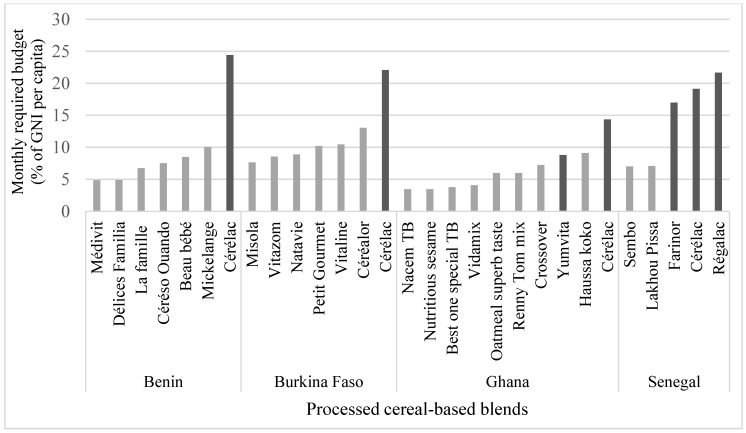
Estimation of the budget required to feed a 6–23 month old child assuming consumption of 50 g of infant flour/day [18] expressed as the percentage of the monthly Gross National Income (GNI) per capita. Darker bars are for products that are imported or produced under license from a large multinational enterprise. (TB, Tom Brown).

Using the mean price of each flour, the budget needed to feed a 6–23 month-old child was calculated assuming consumption of 50 g of PCBB per day and expressed as part of the gross national income (GNI) per capita [17] (Table 1). Using industrial products imported or produced under license from a multinational enterprise and called “imported products”, thereafter, this budget represented 9% to 24% of the GNI per capita (Figure 1). Found in all the four countries surveyed, Cerelac™ was produced by a multinational enterprise in Ghana and exported to Benin, Burkina Faso, and Senegal, allowing comparison from one country to another. The budget it represented ranged from 14% to 24%, depending on the GNI per capita of each country (Table 1). When using a locally-produced PCBB, the budget required to feed a child was much lower than with imported products, representing 5% to 13% of the GNI per capita. 

### 3.3. Ingredients Used and Proximate and Mineral Composition of the PCBBs 

All the samples collected contained one to several cereals and one or more legumes as a source of protein, as well as lipids, such as soybeans or groundnuts (Table S1). Sesame was another local source of lipid that was sometimes used. Among cereals, maize was the most commonly used (18 out of 32 products), while millet ranked second (14 products). Among oil crops, soybean was used in 20 products and groundnut in 15 products. Sugar and salt were found in, respectively, 20 and 8 products. Milk powder was added in 11 out of 32 products. Only 18 products included a vitamin and mineral (VM) premix. Extrusion cooking or the addition of a source of amylase (industrial or from malt), known to be two methods that allow an increase in energy density [19], was only used for the production of 14 flours. 

Proximate and mineral composition of the 32 PCBBs was determined and results were compared to the Codex Alimentarius specifications of Codex Stan 074-1981, Rev. 1-2006 [15] and Codex CAC/GL 8-1991 rev. 2013 [14] (Table 3). The 32 collected samples had very different nutrient contents due to the types and proportions of ingredients used. Protein contents complied with the *Codex Alimentarius* specifications in most products: 24 products according to Codex CAC/GL 8-1991 rev. 2013, the protein contents of the remaining eight products being above the recommended range; 30 products according to Codex STAN 074-1981 rev. 2006, the protein content of the remaining two products being just a little below the recommended range. However, the fat contents were below, and often far below, the level recommended in the *Codex Alimentarius* specifications in 24 out of 32 PCBBs. 

Regarding mineral composition, iron contents varied considerably, ranging from 2.9 to 83.3 mg/100 g DM; 24 PCBBs complied with the recommended iron content, even when no premix was added in nine products. Zinc contents were, mainly, very low, with only nine flours complying, including two out of four imported products. The copper contents were lower than the recommended amount in 12 local and imported PCBBs. Only three samples from Ghana contained sodium above the recommended maximum level, indicating that too much salt had been added. Surprisingly, two (50%) imported PCBBs also had low contents of magnesium, copper, and zinc in two of them. Finally, the calcium and phosphorus contents of all the samples were below the recommended values (except for one, I26, for calcium) even if they were closer to these values in fortified products from Burkina Faso and imported. Overall, two products (BF27 and BF29) from Burkina Faso met eight out of the 10 nutritional specifications studied and five products (three from Burkina Faso, BF28, BF30, and BF31, and two from Ghana, Gh12, and Gh16) met seven specifications. These seven products could be considered as having a satisfactory nutritional value.

### 3.4. Compliance of Labelling Information on the Packaging 

Information on the packaging was assessed using the stipulations reported in Quinn et al. [20] and in the report of the World Health Assembly on mother, infant, and young children nutrition [21]. Only 22 of the 32 samples collected had the nutritional composition and the energy value indicated on their packaging (Table 4). 

Only 20 products provided instructions for appropriate and safe storage or a batch number. Thirteen samples displayed a national certification on their packaging. Concerning the 13 products that displayed a national certification label, the regulations applied were applicable to every type of food product intended for all consumers. Regulations displayed on the products’ packaging are DANA in Benin, Food & Drugs and “Standard Board” in Ghana or “Authorization FRA” in Senegal. No certification label was provided on the packaging of Burkinabe products as there was no certification organism at the time of sampling, although there was a specific standard for PCBBs in this country [22]. Only eight products out of the 32 proposed a daily ration per serving and four of them were those produced by or under license from a multinational enterprise. More seriously, only eighteen products stated an appropriate age of introduction (six months), and one of them used the wording “weaning babies (six months and above)”, which is not appropriate as it could mean breastfeeding cessation. Four products stated the age of introduction as three or four months, whereas the recommended age of introduction is six months (World Health Assembly resolution 54.2. 2003). Only one product asserted the importance of exclusive breast feeding up to the age of six months and only seven encouraged continued breastfeeding up to two years old and beyond. In this respect, four products mentioned that the flour ‘cannot replace breast milk’. Depending on the law applied in the country of sale, pictures of babies may or may not be permitted. Among the eight products showing pictures of babies on the packaging, two of them represented a baby with none of the physical or developmental signs reached after six months and the baby shown was more likely under six months old.

## 4. Discussion

The present study shows that only 22% (7 out of 32) of PCBBs collected in four countries in West-Africa could be classified as nutritionally satisfactory. Compared with the data presented by Trèche and Mbomé [9], clearly progress has been made in the formulation of locally produced PCBBs. There is, especially, a greater use of a vitamin and mineral premix and a more pronounced variety of local products. However, the contribution of locally produced PCBBs to complementary feeding remains very low and deserves encouragement. The results of this study also show that further progress is urgently needed, both in terms of improving nutritional quality and labelling of the products. 

The sampling realized in this study, made on a convenient basis, was neither comprehensive nor representative for the whole four countries, but only representative for the surveyed areas. Notwithstanding, these surveyed areas represented large and medium cities, and urban and rural zones, and corresponded to important portions of the population in each country. The quantities of PCBBs produced monthly were 21 tons by five producers in Benin, 11 tons by six producers in Burkina Faso, and 19 tons by six producers in Ghana (Table 2). Keeping in mind that results of this study are not comprehensive, the quantities reported by the local producers surveyed would represent merely 0.5% to 2.5% of the estimated amount required to feed 6 to 24 month-old children at the national level (Table 1). Thus, this local production would be highly insufficient to feed infant and young children at a national level. 

The surveys conducted at points of sale showed that imported PCBBs were more often available than local products. Previous studies carried out in Senegal and Ghana also showed that the market for complementary foods was dominated by imported products even though Ghana had a wide variety of locally produced PCBBs [23]. In our study, these imported products were two to three times more expensive than local products and were, consequently, not accessible to the majority of the population (Figure S1). Thus, there was a risk that they were used sporadically or over-diluted, since most people could not afford to use them regularly.

Regarding the ingredients used by local producers for processed cereal-based blends, maize, millet, soybean, and groundnut were widely used, which was not surprising as these raw materials are locally produced in the four countries and available throughout the year. Ingredients, such as sugar and salt, were not always mentioned by the producers or on the packaging, particularly, in Ghana, even though they are one way to improve palatability. Among these products, however, three locally produced PCBBs had a very high sodium content, which was above the Codex recommendation, suggesting, as a matter of fact, that ingredients, such as salt or sodium bicarbonate, had been added. Only a few producers added milk powder, which is usually considered to have a positive effect on linear growth through stimulation of the IGF-1 growth factor [24]. Also, in many samples, the main source of fat was only soybean (Table S1) and was used in insufficient proportions, as shown by the low fat content of most products (Table 3). This low fat content, below the Codex recommendation, was also mentioned previously [12]. Producers should, thus, increase the proportion of oilseeds or, if not possible, add oil, although the impact on the conservation of the product would need to be evaluated and could require reducing the recommended maximum storage time. On the contrary, protein content was sufficient in almost all products, but the final proportion of the amino acids and the digestibility of the proteins used would also need to be evaluated. Nevertheless, it is known that soybean and milk proteins are of satisfactory quality and, ideally, complement cereal proteins, especially regarding lysine content. 

Our results also revealed that there were still some not fortified products among the locally produced PCBBs. Some local products, even when not fortified, could include high iron contents, but this was most probably due to contamination caused by milling devices or soil [25]. Infant and young children have high micronutrient requirements due to their rapid development and growth. As they have limited gastric capacity, they should receive a diet mainly composed of nutrient-dense foods [18]. At an individual level, this would be possible by providing to the child a highly diversified diet, including animal source foods. However, such diets have an elevated cost and, at the population level, this would not be compatible with the purchasing power of the majority of households. Indeed, micronutrient deficiencies are widespread, with well-known deleterious consequences for children’s nutritional status and health [26]. Therefore, it is highly advisable that commercial complementary foods are actually fortified with vitamins and minerals. The main constraint is that these premixes are manufactured by international companies and local small and medium sized enterprises must necessarily import them, with minimum quantities imposed. As a result, very small companies (Class III) are often unable to manage this import. In addition, even when fortification was practiced by a noticeable number of producers, many locally produced PCBBs were not adequately fortified. It should also be noticed that imported products did not have adequate zinc, calcium, phosphorus, or magnesium contents. This suggests that PCBB formulation and VM Premix needs to be adjusted to reach the recommended levels to meet the nutrient needs of infants and young children. However, it must be recognized that it is not easy to identify nutritional targets for such products, which requires being aware of current international (or national if any) recommendations and making hypotheses about realistic meal portion sizes and the daily meal number.

Most local products, excluding three that were instant, had to be cooked in water before consumption and their manufacture mainly relied on mechanical unit operations, such as sorting, winnowing, washing, drying, dehulling, and roasting. The first three processes enable the removal of damaged seeds and impurities, whereas dehulling removes seed husks, thus, partially removing fibre, phytates, tannin, and other phenolic compounds, which jeopardize the nutrient bioavailability [27]. Roasting improves digestibility and the organoleptic qualities of the food, as well as reducing microorganisms [14]. However, these dehulling and roasting steps were not applied by all producers. Extrusion cooking, a process combining high temperature, pressure, and shear rate, makes it possible to manufacture instant products. This process reduces the microbial load and the amount of trypsin inhibitors and also makes it possible to obtain a high energy density porridge through partial starch dextrinization [28]. Among the local producers interviewed, only four used the extrusion cooking process, but two others said they produced instant products, although they did not apply extrusion cooking. A process to increase energy density was applied in only 14 products out of 32, although it has been shown to significantly increase energy and nutrient intakes [29]. All these statements demonstrate the importance of supporting local producers who are willing to engage in the production of PCBBs for infant and young children, and training them in the use of the most favourable methods of formulation and processing of cereal-based blends. Some surveyed producers reported some of the difficulties they encounter, such as running out of raw materials, including maize and millet, during the lean season, as well as storing the raw materials in a dry place during the rainy season. Other difficulties encountered were obtaining adequate packaging materials at an acceptable cost, the increasing cost of raw materials, power cuts, and competition with imported products. However, most of them were not aware of all the recommendations surrounding the production of PCBBs. The improvement of formulation, way of processing, and packaging of locally produced PCBBs would probably require an increase in sales price so that the production remains profitable. This asks the question of subsidizing the locally produced PCBBs to maintain its affordability. 

When it comes to marketing and compliance with the international code, the labelling of most PCBBs collected lacked information and showed frequent inconsistencies between their composition and the information printed on their labels, as previously outlined [12]. Some locally produced PCBBs included the word “*enriched*” on the label even when no vitamin and mineral premix had been added. Some PCBBs’ labels also included errors and misleading allegations like “*recommended by WHO*” and whimsical advertisements like “*relieves the symptoms of menopause thanks to oestrogens contained in it*” or “*rejuvenates old people*”! Finally, some PCBBs’ labels showed a lack of compliance with international recommendations for complementary feeding practices (age of introduction, advising daily portion, continuation of breastfeeding, etc.). This was also pointed out for complementary foods commercialized in Senegal and other LMICs [30]. This underlines the need for a specific regulation for complementary foods for 6–23 month-old children. In Ghana [6,31] and Senegal [32], no specific standard or regulation exists for complementary foods. Masters et al. [12,23] reported that complementary foods produced in Ghana or in LMICs had variable nutritional values and suggested that a program aimed at regulating the quality of complementary foods could have a positive impact on the local market, which would then be more competitive and better satisfy young children’s nutritional needs. Regarding Benin, the decree n° 97–643 [33] stipulates that breast milk substitutes and complementary foods commercialized in the country must meet the standards recommended by the Codex Alimentarius and be subject to quality control by the Directorate of Food and Applied Nutrition (DANA). Burkina Faso is a country in which recent projects aimed at the improvement of infant and young child feeding have been implemented, for example, through the Nutrifaso programme, which supports the formulation and the production of adequate complementary foods. In this country, a national standard detailing the desirable composition of PCBBs has also been adopted by the government [22].

## 5. Conclusions and Recommendations

Surveys of producers and points of sale in selected rural and urban areas in Benin, Burkina Faso, Ghana, and Senegal showed that the quantity of locally produced PCBBs and availability at point of sale were limited, with the market being dominated by imported products. Yet, local products are most often cheaper and, consequently, more accessible to the populations and their production should, therefore, be encouraged. However, based on the characteristics of the 32 samples collected and reviewed in this study, only seven products showed satisfactory nutritional composition. This shows that, although progress has been made in the last thirty years, poor nutritional quality and inappropriate labelling of numerous PCBBs sold in low and middle income countries remains a serious problem today. This highlights the compelling need to enact not only specific regulations for marketed complementary foods for children aged 6 to 24 months at national or subregional levels and accompanied by the establishment of national control bodies, but also the importance of providing technical and financial support to producers.

## Figures and Tables

**Table 1:** Rough estimation of the monthly needs in fortified PCBBs^a^ used as complementary foods in four West African countries.

Country	Annual GNI Per Capita (€) ^b^	Estimation of the Number of 6–23 Months Children (2016) ^c^	Estimated PCBBs Needs (tons/month) ^d^
Benin	759	524,000	800
Burkina Faso	578	878,000	1400
Ghana	1338	1,320,000	2000
Senegal	904	734,000	1200

^a^PCBBs= processed cereal-based blends; ^b^ GNI = Gross National Income. GNI/capita/year in 2015 [17] and converted from $ to € using a change of 0.904 (average change from January 2015 to January 2017); ^c^ calculated from population pyramids’ data of 2016 (https://populationpyramid.net/fr/); ^d^ these estimations were calculated taking into account a daily average consumption of 50 g of PCBB/day for 6–23 month-old children [18] and values were rounded.

**Table 2:** Classification of producers surveyed according to the estimated quantity of PCBBs produced.

Class	Name of Enterprise	Brand Names of PCBB Produced	Mean Quantity Produced (tons/month)	Country of Production	Countries of Exportation
Class I (> 3 tons, > 2000 IYC)	PKL	Farinor (3 varieties) Nutribon (4 varieties)	17.5	Ivory Coast ^a^	Cameroun, Ghana, Senegal, Togo
	Blessed Child Foods	TB, Oatmeal superb taste, Cereal plus, Rice mix, Multicereal, Nutrimental, Brown Rice	15.0	Ghana	None
	CVSFEB	Beau bébé (1st and 2nd age), Pépite d’or	7.0	Benin	Togo, Niger
	Agrotechnic	Mickelange	6.0	Benin	Niger
	UBETA/DANA	Céréso Ouando, Rimalait	6.0	Benin	Burkina Faso, Niger, Togo
	Misola Burkinabè Association	Misola	4.0	Burkina Faso	Benin, Burkina Faso, Mali, Niger, Senegal ^b^
	SODEPAL	Vitaline	3.5	Burkina Faso	None
Class II (0.6 to 3 tons, 400 to 2000 IYC)	Elssy Kess Homefresh	Haussa koko	1.8	Ghana	None
	JFP	La famille (1st and 2nd age)	1.5	Benin	None
	ECOPRIX	Petit Gourmet	1.2	Burkina Faso	None
	Renny Foods	TB, Renny Tom cereal legume mix	1.0	Ghana	None
	FASO RIIBO	Natavie	0.8	Burkina Faso	None
	Burkina Agricole	Vitazom	0.7	Burkina Faso	None
	CTRAPA	Céréalor	0.6	Burkina Faso	None
Class III (< 0.6 ton, < 400 IYC)	Vidamix Foods	Vidamix roasted cereal mix (3 var)	0.4	Ghana	None
	Justibek Foods	Nutritious sesame	0.4	Ghana	None
	Sembo	Sembo, Lakhou Pissa	0.4	Senegal	None
	Medic Group Services	Médivit with soybean (from 3 mo), Médivit without soybean (from 6 mo)	0.2	Benin	None
	Crossover	Crossover TB (5 varieties)	0.1	Ghana	None
	NA	Delices Familia	NA	Benin	NA

IYC, Infants and Young Children; TB, Tom Brown; NA, Not available. ^a^ The PCBB ‘Farinor’ is produced in Ivory Coast, but the sample was collected in Senegal. ^b^ AB Misola Association, based in Burkina Faso, has installed production units in each of the countries listed as countries of exportation.

**Table 3:** Proximate and mineral composition (per 100 g dry matter -DM) of the 32 PCBB samples.

	PCBB Code	Proximate Composition (g/100 g DM)		Mineral Composition (mg/100 g DM)							
		Protein	Fat	Fe	Zn	Ca	Mg	Na	Cu	P	Mn
Without VM Premix	Be7	**11.5**	6.7	**7.1**	2.8	31	**121**	**0.8**	0.29	300	0.9
	Be8	**10.1**	4.9	**13.8**	3.1	12	**110**	**2.9**	0.25	297	0.7
	Gh10	**13.5**	9.1	**13.4**	4.1	48	**128**	**2.1**	**0.49**	279	**1.5**
	Gh11	**14.9**	**12.2**	4.1	2.7	44	**126**	**3.7**	**0.36**	277	**1.3**
	Gh12	**16.7**	7.4	**83.3**	**4.4**	93	**151**	**1.7**	**0.61**	351	**2.4**
	Gh13	**18.4**	7.8	**20.3**	3.8	175	**122**	517	**0.46**	373	**2.3**
	Gh14	**16.6**	**13.5**	6.0	3.0	42	**128**	**1.5**	**0.38**	285	1.1
	Gh15	**21.8**	7.7	**12.8**	3.0	138	**132**	487	**0.59**	316	**2.8**
	Gh16	**18.3**	**9.6**	**49.4**	3.5	93	**143**	**2.8**	**0.59**	393	**3.2**
	Gh17	**19.7**	8.8	**6.2**	3.0	88	**115**	**1.9**	**0.57**	262	**2.1**
	Gh18	**15.2**	**11.9**	6.1	3.4	137	**134**	**9.7**	**0.44**	345	**1.3**
	Gh19	**10.2**	2.8	**25.6**	2.7	18	**121**	**3.0**	0.30	255	**1.5**
	Se21	**14.9**	7.9	4.8	3.0	24	**116**	**4.8**	**0.38**	260	**1.4**
	Se22	**22.2**	**18.8**	4.2	3.0	62	**153**	793	**0.52**	328	**1.8**
With VM Premix	Be1	**9.2**	5.5	2.9	2.3	13	**81**	**168**	0.23	208	0.7
	Be2	**11.7**	7.1	4.1	2.5	42	**93**	**228**	0.29	236	0.8
	Be3	**15.9**	6.6	**28.2**	2.8	81	**116**	**258**	**0.43**	282	**1.4**
	Be4	**17.3**	6.8	**28.3**	2.7	93	**126**	**184**	**0.45**	318	**1.4**
	Be5	**7.7**	4.6	**9.2**	1.3	77	49	**48**	0.18	149	0.6
	Be6	**14.3**	4.9	**7.3**	2.9	56	**122**	**1.0**	**0.36**	309	1.1
	BF27	**15.5**	**10.4**	**11.7**	**10.8**	223	**151**	**342**	**0.74**	263	**2.0**
	BF28	**15.3**	9.1	**13.0**	**10.4**	338	**132**	**337**	**0.59**	227	**1.6**
	BF29	**16.6**	**11.5**	**16.6**	**13.2**	417	**135**	**315**	**0.79**	109	**1.9**
	BF30	**15.2**	8.3	**18.0**	**11.9**	381	**160**	**196**	**0.81**	246	**2.3**
	BF31	**13.3**	7.4	**13.7**	**10.4**	272	**107**	**304**	**0.55**	150	**1.4**
	BF32	**10.7**	7.5	**8.4**	3.5	137	54	**91**	0.23	150	0.8
	BF33	**8.5**	5.8	5.0	3.9	155	55	**213**	0.20	159	0.3
	Se20	**12.0**	2.1	**26.1**	**20.3**	384	50	**101**	**1.37**	309	3.0
	I24	**15.4**	3.8	**11.9**	**8.2**	456	50	**115**	0.06	340	0.3
	I25	**9.9**	6.1	**33.2**	1.9	400	60	**116**	0.19	372	1.3
	I26	**11.6**	5.4	**29.5**	2.1	**566**	**65**	**107**	0.23	463	1.3
	I34	**14.4**	**9.6**	**8.9**	**5.5**	414	37	**91**	0.06	270	0.3
Codex specifications ^a^	Min	6.5 ^d^–8.6 ^e^	9.6 ^b,d^	6.2 ^c^	4.4 ^c^	536	64	--	0.36	493	1.3
	Max	16.1 ^d^–23.7 ^e^	19.4 ^d^	-	-	-	-	430	-	-	-
**Number of compliant products**		**24 ^d^–30 ^e^**	**8**	**24**	**9**	**1**	**23**	**29**	**20**	**0**	**18**

Correspondence between brand names and samples numbers was voluntarily not provided for confidential reasons. DM, Dry Matter; VM Premix, vitamin and mineral premix; Be, Benin; BF, Burkina Faso; Gh, Ghana; Se, Senegal; I, Imported or produced under license of a multinational enterprise (lines highlighted in grey). Values in bold character are those complying with Codex Alimentarius specifications. ^a^ The nutritional specifications were calculated assuming a DM content of 93% (average of all 32 samples DM values), an energy value of 430 kcal/100 g DM, and a daily portion of 50 g; ^b^ minimal amount of fat required was calculated assuming that at least 20% of energy should derive from fat; ^c^ assuming medium levels of bioavailability of 10% for iron and 30% for zinc; ^d^ Codex CAC/GL 8-1991 Rev. 2013 [14]; ^e^ Codex STAN 074-1981 rev. 2006 [15].

**Table 4:** Criteria used to evaluate the adequacy of the labelling of PCBBs marketed as complementary foods for 6–23 month-old children.

Recommendations Figuring in the Codex Stan 074 Rev. 2006		Number of Compliant Products Out of 32 (%)
Information that MUST figure on the packaging	Ingredients	29 (91)
	Nutritional composition and energy	22 (69)
	Provides instructions for appropriate preparation and use	29 (91)
	Provides instructions for safe and appropriate storage	20 (63)
	Batch number	20 (63)
	Expiry date	31 (97)
	Producer’s name	29 (91)
	Producer’s address	32 (100)
	National certification ^a^	13 (41)
Recommendations Based on the International Code and WHA Resolutions		Number of Compliant Products Out of 32 (%)
TO DO	Proposes a daily ration per serving	9 (28)
	Specifies an appropriate age of introduction (from 6 months)	18 (56)
	If pictures are permitted by national laws, pictures of babies must show babies older than 6 months (with physical or developmental milestone reached after 6 months)	7 (22)
	States the importance of exclusive breastfeeding till 6 months	1 (3)
	Encourages continued breastfeeding up to 2 years old and beyond	8 (25)
	With the indication ‘‘Can Not replace breast milk’’	5 (16)
Recommendations based on the International Code and WHA resolutions		Number of NOT Compliant Products Out of 32 (%)
NOT TO BE DONE	States an age of introduction less than 6 months	4 (13)
	If pictures are permitted by national laws, pictures of babies must not show a baby) less than 6 months old (ex: baby lying on its belly)	4 (13)
	Mentions whimsical or misleading allegations	3 (9)

WHA, World Health Assembly. ^a^ The four countries where the surveys were conducted do not have a national regulation specifically regarding complementary food intended for young children. Thus, the term ‘National Certification’ represents here regulations regarding all types of food intended for consumers of any age.

## Supplementary Materials

The following are available online at http://www.mdpi.com/2072-6643/10/6/785/s1, Figure S1: Mean prices of PCBBs (in euro/kg) according to the country where they were collected. Prices were collected at POS during the period from April to June 2016. TB: Tom brown. Darker bars are for products that are imported or produced under license from a large multinational enterprise; Table S1: Basic ingredients used in the 32 collected samples of PCBBs.
