# Supplementary material for: Adequacy of Some Locally Produced Complementary Foods Marketed in Benin, Burkina Faso, Ghana, and Senegal
Source: Nutrients. 2018 Jun 18;10(6):785. doi: 10.3390/nu10060785 (PMC6024696; doi:10.3390/nu10060785)
Supplement: Supplementary file 1 [file nutrients-10-00785-s001.pdf]

Table S1. Basic ingredients used in the 32 collected samples of PCBBs.

Country of origin	PCBB commercial name	Flour type[a]	Basic ingredients			Process used to increase energy density	VM Premix[a]
			Carbohydrate source	Oleaginous & Legumes	Other		
Locally processed PCBBs							
Benin	Beau bébé (1st age)	C	maize, rice	soybean germ	sugar, fat	malted cereals	yes
	Beau bébé (2nd age)	C	maize, rice, tubers	soybean	sugar, fat	none	yes
	Céréso Ouando	I	maize, sorghum	soybean	sugar	extrusion cooking	yes
	Délices Familia	C	maize, millet	_	salt	none	no
	La famille (1st age)	C	maize, millet	_	milk, sugar, vanilla	none	yes
	La famille (2nd age)	C	maize, millet, sorghum	soybean	milk, sugar, vanilla	none	yes
	Médivit (from 3 mo)	C	maize, millet, rice, fonio	soybean	_	none	no
	Mickelange (2nd age)	I	maize, millet, cassava	_	milk, egg, sugar, salt	extrusion cooking	yes
Burkina Faso	Céréalor	C	yellow maize	groundnut, soybean	milk, sugar, iodized salt	industrial amylase	yes
	Misola	C	millet	groundnut, soybean	sugar, iodized salt	industrial amylase	yes
	Natavie	C	millet, sorghum	groundnut, soybean	sugar, iodized salt, baobab fruit	industrial amylase	yes
	Petit Gourmet	C	white maize	groundnut, soybean, sesame	sugar, iodized salt	industrial amylase	yes
	Vitaline biscuitée	C	maize, wheat	groundnut	milk, sugar, bicarbonate sodium, citric acid, dipotassium phosphate	none	yes
	Vitaline Instantanée	I	maize	groundnut	milk, sugar	extrusion cooking	yes
	Vitazom	C	white maize	groundnut, soybean, cowpea	sugar, iodized salt, vanillin	industrial amylase	yes
Ghana	Best one special TB	C	rice, wheat	soybean	_	none	no

Country of origin	PCBB commercial name	Flour type[a]	Basic ingredients			Process used to increase energy density	VM Premix[a]
			Carbohydrate source	Oleaginous & Legumes	Other		
	Crossover – millet & wheat TB	C	millet, wheat	groundnut, soybean	sugar	none	no
	Crossover – wheat & soybean TB	C	wheat	soybean	sugar	none	no
	Homefresh Haussa koko	C	millet, sorghum	_	spices	none	no
	Nacem TB	C	maize	groundnut, soybean	_	none	no
	Nutritious sesame	C	maize, millet	groundnut, soybean, sesame, flax seeds,	_	none	no
	Oatmeal superb taste	C	millet, sorghum, oat	soybean	_	none	no
	Renny Tom cereal legume mix	C	rice, wheat	groundnut, soybean, beans	_	none	no
	Vidamix - maize	C	maize	groundnut, soybean	_	none	no
	Vidamix - millet	C	millet	groundnut, soybean	_	none	no
Senegal	Sembo	C	maize, millet, rice	groundnut, cowpea		none	no
	Sembo Lakhou Pissa	C	millet	groundnut, cowpea	smoked dry fish, bissap	none	no
PCBBs imported or produced under license of a multinational enterprise							
Ivory Coast[b]	Farinor-rice	I	rice	soybean	milk, sugar, flavorings	extrusion cooking	yes
Brasil[b]	Régalac-5 cereals	I	maize, rice, wheat, barley, oat flakes	_	milk, sugar, vegetable fat, inulin, vanilla flavor	extrusion cooking	yes
Brasil[b]	Régalac-wheat, milk, 3 fruits	I	wheat	_	milk, sugar, vegetable fat, banana, papaya, apple, inulin, vanilla flavor	extrusion cooking	yes
Ghana[c]	Cerelac-my 1st wheat cereal	I	wheat	_	milk, sugar, salt, palm olein, maltodextrins, vanillin, bifidus culture	extrusion cooking	yes

Country of origin	PCBB commercial name	Flour type[a]	Basic ingredients			Process used to increase energy density	VM Premix[a]
			Carbohydrate source	Oleaginous & Legumes	Other		
Ghana[d]	Yumvita	I	maize, wheat	_	milk, sugar, maltodextrins, vegetable and fish oil, vanillin	extrusion cooking	yes

[a] The flour type and VM Premix addition were determined according to the recommended mode of preparation on the packaging and information collected among producers. VM, Vitamins and Minerals; I, Instant; C, to be cooked; TB, Tom brown; [b] Imported into Senegal; [c] Produced in Ghana by Nestlé - a Swiss multinational company, and imported into Senegal; [d] Produced in Ghana, under license for Promasidor in Switzerland.

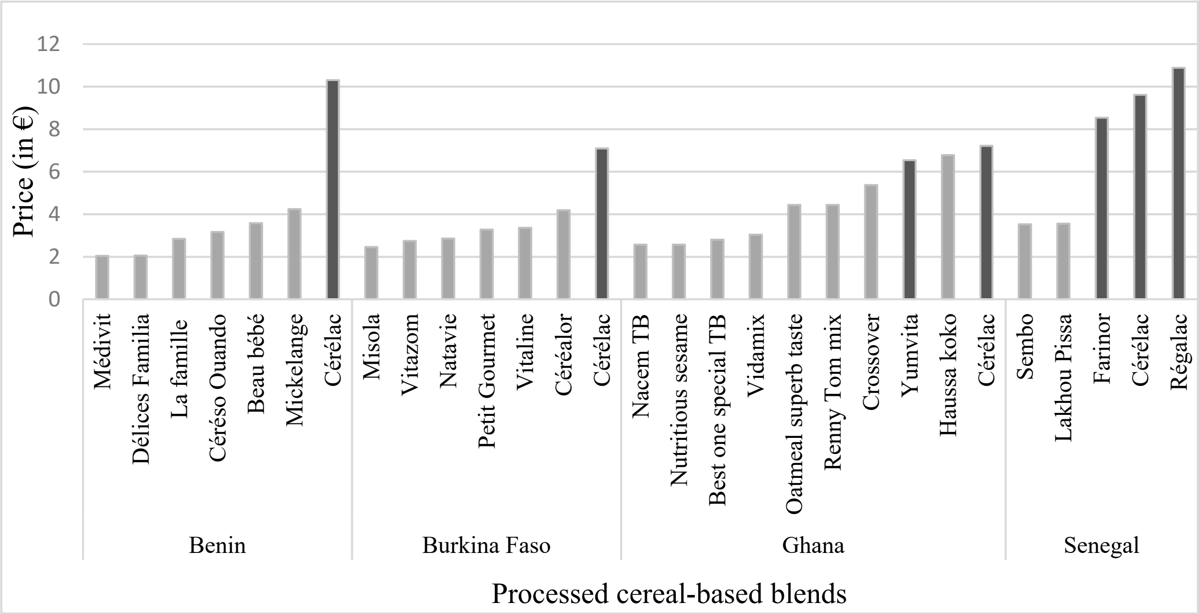

Figure S1: Mean prices of PCBBs (in euro/kg) according to the country where they were collected. Prices were collected at POS during the period from April to June 2016. TB : Tom brown. Darker bars are for products that are imported or produced under license from a large multinational enterprise.
